# Thermal Ageing of Black Garlic Enhances Cellular Antioxidant Potential Through the Activation of the Nrf2-Mediated Pathway

**DOI:** 10.17113/ftb.63.01.25.8474

**Published:** 2025-03

**Authors:** Shan-Shan Yu, Yung-Lin Chu, Yen-Chen Tung, Zheng-Yuan Su

**Affiliations:** 1 Department of Bioscience Technology, Chung Yuan Christian University, Taoyuan City 320314, Taiwan, Republic of China; 2 Department of Food Science, National Pingtung University of Science and Technology, Pingtung County 912301, Taiwan, Republic of China; 3 Department of Food Science, National Ilan University, Yilan County 260007, Taiwan, Republic of China

**Keywords:** garlic, Maillard reaction, liver, Nrf2, oxidative stress, antioxidant properties

## Abstract

**Research background:**

Oxidative stress plays a crucial role in different diseases, including chronic hepatitis, cirrhosis and liver cancer, which are a major cause of mortality worldwide. Liver cell injury resulting from oxidative stress contributes to the development of these diseases. Garlic is known for its diverse physiological activities, and black garlic, produced by thermal ageing of raw garlic, has attracted attention due to its biological properties.

**Experimental approach:**

This study investigates the hepatoprotective potential of black garlic prepared in an electric cooker. The study includes mass loss, browning index, free amino acids, free-reducing sugar content, total phenolic compounds and DPPH radical scavenging activity. Additionally, the sensory evaluation shows a preference for the black garlic sample over raw garlic. The study also investigates the activation of Nrf2-ARE pathway in HepG2-C8 cells and evaluates the protective effect against H_2_O_2_-induced damage.

**Results and conclusions:**

The results indicate that black garlic lost mass, possibly due to water loss and the Maillard reaction, which led to an increase in the browning index and a decrease in free amino acids. However, the content of free reducing sugars increased. After 14 and 21 days, the total phenolic content of black garlic increased and its ability to scavenge DPPH radicals improved. Significant activation of the Nrf2-ARE pathway was observed in HepG2-C8 cells. Sensory evaluation showed a preference for the 14-day aged black garlic. The Nrf2 pathway can be effectively activated in HepG2 cells by a 14-day aged black garlic extract, resulting in protection against H_2_O_2_-induced damage.

**Novelty and scientific contribution:**

Our research shows the significant effect of thermal ageing on black garlic and highlights its enhanced antioxidant properties. A simple approach has been developed to prepare black garlic that is more effective, healthier and can potentially be used to protect the liver and treat diseases related to oxidative stress.

## INTRODUCTION

An imbalance of reactive oxygen species (ROS) in cells or tissues causes oxidative stress, which leads to a disruption of physiological activities (*1*). High amounts of reactive oxygen species, including superoxide radicals, hydrogen peroxide, hydroxyl radicals and singlet oxygen, can damage the cell structure or DNA and impair normal cellular functions (*2*). The transcription factor nuclear factor erythroid 2-related factor 2 (Nrf2) regulates cellular redox reactions and controls the expression of different antioxidant and detoxification enzyme genes. It acts as a defence system in the body and protects normal cells from injury caused by oxidative stress and toxic substances (*3*). In the cytoplasm, Nrf2 is bound to Keap1 (kelch-like ECH-associated protein 1) under normal conditions, resulting in an inactive state. Nrf2 dissociates from Keap1 and translocates into the nucleus to form a heterodimer with Maf proteins when exposed to oxidative stress (*4*). The complex binds to antioxidant response elements (AREs) and activates genes that encode different antioxidant and detoxification enzymes, such as haem oxygenase 1 (HO-1), NAD(P)H:quinone oxidoreductase-1 (NQO1), and UDP-glucuronosyltransferases (UGTs) (*5*). These enzymes play a crucial role in mitigating cellular damage caused by toxic substances and free radicals, while also exerting inhibitory effects on the carcinogenic process (*6*).

The liver is the main digestive organ of the human body and is responsible for various functions of detoxification and metabolism, including the metabolism of nutrients, homeostasis of lipids and cholesterol, and regulation of endocrine activities (*7*). Exposure to substances like drugs, alcohol or toxins can cause oxidative stress in liver cells, which can lead to the development of liver diseases such as alcoholic fatty liver, non-alcoholic fatty liver and chronic hepatitis (*8*). Peroxides generated by mitochondria and peroxisomes can induce lipid peroxidation and activate hepatic stellate cells or cause hepatocyte death (*8*). However, activation of Nrf2 can increase the activity of cellular antioxidants and protect liver cells from oxidative stress (*6*). Phytochemicals, such as the various phenolic compounds found in plants, have a variety of physiological effects that include anti-inflammatory, antioxidant and anti-cancer activities (*9*). Some studies have shown that phytochemicals can protect the liver by activating the Nrf2 pathway (*10*).

Garlic (*Allium sativum*) is a plant that belongs to the Amaryllidaceae family and has long been used both as a culinary ingredient and as a medicinal plant. Organosulfur compounds, saponins and phenols are the bioactive compounds valued for their multiple health benefits, including anti-inflammatory, antioxidant and anticancer properties (*11*). Although garlic is generally considered healthy, some people may find it difficult to tolerate its pungent taste. Black garlic is a processed food derived from fresh garlic that undergoes an enzyme-free browning reaction called the Maillard reaction under high temperatures and humidity (*12*). The aroma of black garlic is more subdued and it contains bioactive compounds, including phenolic, flavonoid and sulfur compounds (*13*). The sulfur compounds found in fresh garlic are γ-glutamyl-*S*-alk(en)yl-l-cysteine and *S*-alk(en)yl-l-cysteine sulfoxides, the primary precursors of *S*-allylcysteine (*14*). *S*-allylcysteine and hydroxymethylfurfural have been found in black garlic (*15*). To produce black garlic, fresh garlic is exposed to a high-temperature and high-humidity environment, which leads to a non-enzymatic browning reaction known as the Maillard reaction (*16*). The garlic cloves turn brown-black through this process and have a soft texture and a slightly sugary, tangy taste reminiscent of dried fruit.

Black garlic has been found to contain various antioxidant and anti-inflammatory components, including phenolic compounds (*17*). Studies have shown that black garlic extract has the potential to regulate lipid metabolism, reduce insulin resistance and treat chronic diseases (*18*). An incubator with a constant temperature of over 75 °C is used in the production of black garlic, as mentioned in most research papers (*17*, *19*). Scientific studies on the preparation of black garlic using an electric cooker, which usually maintains a core temperature of 50 to 72 °C, are lacking. The aim of this study is to prepare black garlic in an electric cooker and to evaluate the changes in the composition of the samples after several days of thermal ageing. We investigated the ability of black garlic extract to scavenge DPPH radicals and its potential to activate cellular antioxidant response element (ARE)-luciferase activity using HepG2-C8 cells. We also investigated whether black garlic extract can enhance the expression of Nrf2 and downstream antioxidant genes, which could potentially mitigate H_2_O_2_-induced hepatic cell damage.

## MATERIALS AND METHODS

### Materials

Trolox, 2,4,6-trinitrobenesulfonic acid (TNBS), dimethyl sulfoxide, Folin-Ciocalteu reagent, glucose, hydrochloric acid, l-leucine, sodium hydrogen carbonate, sodium carbonate, sodium chloride, 2-amino-2-(hydroxymethyl)-1,3-propanediol (Trizma base) and phenylmethanesulfonylfluoride were purchased from Sigma-Aldrich, Merck (St. Louis, MO, USA). Radioimmunoprecipitation assay (RIPA) buffer was obtained from Cell Signaling (Danvers, MA, USA). Penicillin-streptomycin solution and trypsin were purchased from Thermo Fisher Scientific (Waltham, MA, USA). Dulbecco’s modified Eagle (DMEM) and foetal bovine serum (FBS) were obtained from Gibco (Dublin, Ireland). DPPH was purchased from Alfa Aesar (Haverhill, MA, USA). Gallic acid was from Tokyo Chemical Industry (Tokyo, Japan), while 3,5-dinitrosalicylic acid (DNSA) and methanol were obtained from ECHO Chemical (Miaoli, Taiwan).

### Black garlic preparation

**Fig. S1:**
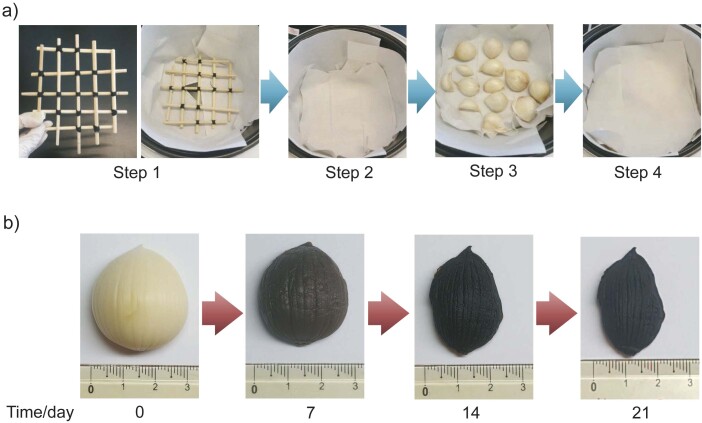
Steps for black garlic preparation: a) thermal ageing process, and b) the effect of thermal ageing on the appearance of garlic. Step 1. Raise the bottom layer using a homemade bamboo rack. Step 2. Spread kitchen paper towels on top. Step 3. Evenly spread out the peeled garlic cloves. Step 4. Cover with another layer of kitchen paper towels. Stack up to three layers and place kitchen paper towels on the top layer. Finally, cover the pot with the lid of the electric cooker and keep it at a warm setting. After ageing for 7, 14 and 21 days, respectively, remove the black garlic

The black garlic was obtained from the market in Xiluo Township, Yunlin County, Taiwan. The thermal ageing process of black garlic is shown in Fig. S1. After peeling the fresh garlic cloves, a bamboo rack was used to lift the bottom layer and prevent direct contact with the bottom of the electric cooker (KH-WA10T; SAMPO, Taoyouan City, Taiwan) (Fig. S1a). Paper towels were placed on the bamboo rack to prevent staining and to ensure that the garlic was evenly distributed without falling into the gaps. Another layer of paper towels was placed on top and the garlic cloves were stacked in the electric cooker. The top layer was covered with kitchen paper towels to prevent condensation from dripping onto the cloves during the process. The electric cooker was then closed with the lid and set to the warm setting. After ageing for 7, 14 and 21 days, the black garlic was collected and stored in sealed bags. The appearance of fresh (day 0) and black garlic is shown in Fig. S1b.

### Water content and mass loss determination

To determine the water content of fresh or black garlic, approx. 1.0 g of the sample was placed in an aluminium dish and dried in a preheated oven at 105 °C (MOV-212F-PK; Panasonic, Osaka, Japan). The sample was removed every 2 h and immediately placed in a desiccator to cool and then weighed at room temperature. The drying and weighing steps were repeated until a constant mass was achieved (ME2002; Mettler Toledo, Columbus, OH, USA). The moisture content (g/100 g) was calculated using the following equation:


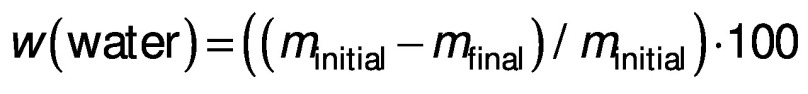
 /1/

where *m*_initial_ is the initial mass of the sample before drying and *m*_final_ is the final mass of the sample after drying. Mass loss (g/100 g) was determined according to the following equation:


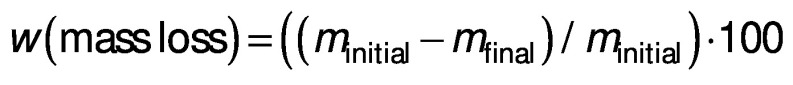
 /2/

### Browning index analysis

The browning index was measured according to the published study (*20*) with some modifications. Fresh and black garlic cloves were homogenised with ddH_2_O at a ratio of 1:10 (Bioprep-24; Allsheng, Hangzhou City, PR China). A volume of 1.0 mL of the homogenised sample was then transferred to microcentrifuge tubes and centrifuged at 17 000×*g* for 10 min at 25 °C (Fresco 17 centrifuge; Thermo Fisher Scientific). After 200 μL of the supernatant were transferred to a 96-well plate, the absorbance was measured at 420 and 550 nm using a BioTek microplate reader (Synergy HT, Winooski, VT, USA). The browning index refers to the difference between the absorbance at 420 and 550 nm.

### Free reducing sugar determination

The homogenised fresh and black garlic cloves were prepared with ddH_2_O at a ratio of 1:10 (Bioprep-24 homogeniser) according to a previously described method (*21*). Subsequently, 1 mL of the sample or glucose standard solution together with 150 μL of ddH_2_O and 250 μL of DNSA reagent, were added sequentially and mixed thoroughly. The reaction mixture was then incubated at 100 °C in a water bath for 10 min. After cooling to room temperature, 200 μL of the reaction mixture were carefully transferred to a clear 96-well plate and the absorbance was measured at 540 nm using a microplate reader (Synergy HT). The relative content of reducing sugars in the samples was determined using the regression equation derived from the standard curve.

### Free amino acid determination

The homogenized fresh and black garlic cloves were dissolved in a solution containing 1% sodium dodecyl sulfate (SDS) according to a previously described method (*21*). In a microcentrifuge tube, 20 μL of the sample or l-leucine standard solution were added, followed by 160 μL of 0.2 M phosphate buffer (pH=8) and 160 μL of 0.1% TNBS solution. The mixture was mixed well and incubated at 50 °C in a water bath for 60 min. After cooling to room temperature, 320 μL of 0.1 M HCl were added to the mixture. Finally, 200 μL of the test sample were transferred to a 96-well plate and the absorbance was measured at 340 nm using a microplate reader (Synergy HT). The relative content of amino acids in the samples was calculated using the regression equation obtained from the standard curve.

### Determination of total phenolic compounds

Fresh and black garlic cloves were homogenised using a ratio of 1:10 of 95% methanol (Bioprep-24 homogeniser). After centrifugation at 17 000×*g* for 5 min (Fresco 17 centrifuge; Thermo Fisher Scientific), the supernatant was collected. Gallic acid was used as a standard. For the assay, 50 μL of the supernatant and 100 μL of 10% Folin-Ciocalteu reagent were mixed in a microcentrifuge tube. Subsequently, 400 μL of 700 mM Na_2_CO_3_ were added to the mixture, mixed thoroughly and left at room temperature for 1 h. A volume of 200 μL of the reaction mixture was then transferred to a transparent 96-well plate and the absorbance was measured at 765 nm using a microplate reader (Synergy HT). The gallic acid content was calculated in milligrams of gallic acid equivalent (GAE) per 100 grams using the following formula:


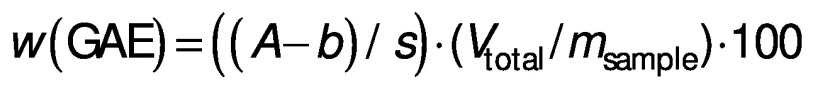
 /3/

where *A* is the absorbance of the sample, *b* is the intercept of the standard curve, *s* is the slope of the standard curve, *V*_total_ is the total volume of the extract prepared from the garlic sample and *m*_sample_ is the mass of the garlic sample used for extraction.

### DPPH radical scavenging assay

The method used in this study was based on an earlier work (*22*). Fresh and black garlic cloves were homogenised using a ratio of 1:10 of 95% methanol (Bioprep-24 homogeniser). Then, 0.5 mL of the centrifuged supernatant was mixed with 1.2 mL of ethanol and 0.3 mL of a 0.5 mM DPPH solution and allowed to react for 20 min. Trolox was used as a positive control. The absorbance of the reaction mixture was measured at a wavelength of 517 nm using a microplate reader (Synergy HT). The DPPH radical scavenging activity was calculated as follows:


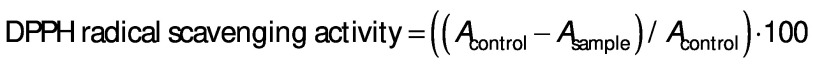
 /4/

where *A*_control_ is the absorbance of the control (DPPH solution without the sample) and *A*_sample_ is the absorbance of the reaction mixture containing the sample.

### Preparation of black garlic water extracts for cell culture

A mass of 30 g of fresh or black garlic was weighed and mixed with 500 mL of distilled water. The mixture was blended until it was well crushed. A magnetic stirrer bar was placed in the beaker containing the mixture and stirred for 1 h (PC-420D stirring hot plate; Corning, Corning, NY, USA). The resulting filtrate was collected and lyophilised to remove water (freeze-drying system 7806031; Labconco, Kansas, MO, USA). The yield of water extracts from fresh garlic and black garlic aged for 7, 14 and 21 days was 28.4, 28.3, 29.3 and 30.2 g/100 g, respectively.

### Cell culture

Human hepatocellular carcinoma HepG2 cells (ATCC HB-8065) and HepG2-C8 cells stably transfected with the ARE-luciferase plasmid were cultured in DMEM medium supplemented with 10% FBS, 3.7 g/mL NaHCO_3_, 50 U/mL penicillin and 50 µg/mL streptomycin. The cells were incubated in a humidified incubator with 5% CO_2_ (MCO-170AIC; Panasonic) at 37 °C to maintain optimal conditions.

### ARE-luciferase activity evaluation

The ability of the samples to induce ARE-luciferase activity was assessed using HepG2-C8 cells that were stably transfected with the ARE-luciferase plasmid according to a previously described method (*23*). HepG2-C8 cells were seeded at a density of 10^5^ cell/mL in a 12-well plate and incubated for 24 h. The cells were then treated with a culture medium containing 100 μg/mL of fresh garlic or black garlic extract for another 24 h. The concentration of 100 μg/mL was chosen because previous studies confirmed that this concentration effectively modulates ARE-luciferase activity in liver cells (*24*). Sulforaphane (SFN) was used as a positive control. After removing the cell culture medium, 55 μL of lysis buffer were added to the cells, which were then stored at -20 °C for 24 h. The cell lysate supernatant was obtained by centrifugation at 17 000×*g* and 4 °C for 10 min (Fresco 17 centrifuge; Thermo Fisher Scientific). The fluorescence intensity was measured using a microplate reader (Synergy HT) according to the instructions of the manufacturer of the Luciferase Assay System (Promega, Madison, WI, USA). The following formula was used to calculate the relative ARE-luciferase activity:


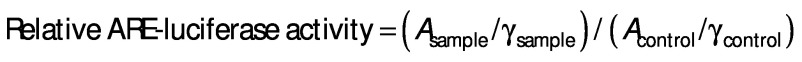
 /5/

where *A*_sample_ and *A*_control_ are the fluorescence intensities of the sample and control, respectively. The protein concentrations of the sample and control (*γ*_sample_ and *γ*_control_) were determined using the bicinchoninic acid (BCA) assay kit (G-Bioscience, Taipei, Taiwan).

### Sensory evaluation

In this study, we used sensory evaluation to determine the preference and acceptance of black garlic with different ageing times. A total of 51 people, 23 women and 28 men, took part in the evaluation. The group consisted of 2 people younger than 20, 41 people between 21 and 25, 4 people between 26 and 30, 2 people between 31 and 35, 1 person between 51 and 55, and 1 person who was 60 years old. A random number was assigned to the samples of fresh and dark garlic. The participants were asked to taste the garlic samples and answer an anonymous questionnaire. On a 9-point scale, they rated the colour, flavour, texture and overall impression of the garlic samples to express their preference. Lemon water was served to cleanse the palate during sampling. After the evaluation of all four garlic samples, the participants were asked to state their personal preference for the samples.

### Elucidation of H_2_O_2_ -induced liver cellular damage

HepG2 cells were seeded at a density of 2·10^4^ cell/100 μL in a 96-well plate and incubated for 24 h (MCO-170AIC incubator; Panasonic). The cells were then treated for 48 h with a culture medium containing various concentrations of 14-day-old black garlic water extract filtered through a 0.22-μm filter. After removing the old culture medium, the cells were treated for 8 h with a culture medium containing 1 or 2 μM H_2_O_2_. The CellTiter 96® AQ_ueous_ One Solution Cell Proliferation (MTS) assay kit (Promega) was used to measure the absorbance at 490 nm (Synergy HT microplate reader) after 1 h of reaction. The absorbance was recorded to calculate the cell viability (% of control group) for each treatment group:


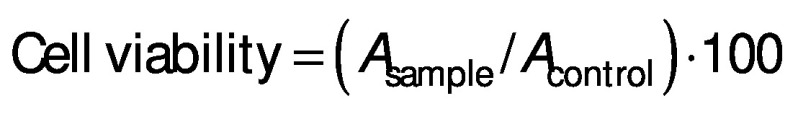
 /6/

where *A*_sample_ is the absorbance of the treatment group and *A*_control_ is the absorbance of the control group.

### Protein expression

HepG2 cells were seeded at a density of 210^6^ cell/10 mL in cell culture dishes containing 10% FBS and incubated for 24 h (MCO-170AIC incubator; Panasonic). They were then treated with different concentrations of 14-day-old black garlic extract for 48 h. After removing the culture medium, the cells were collected, washed and lysed with RIPA buffer (Cell Signaling). The cell lysates were sonicated (Ultrasonic 250 grinder; Hoyu, Taipei, Taiwan) and centrifuged (Fresco 17 centrifuge; Thermo Fisher Scientific) and the supernatant containing 25 μg of protein was used for SDS-PAGE (Mini Gel Tank; Thermo Fisher Scientific) and then transferred to a polyvinylidene fluoride (PVDF) membrane (Trans-Blot® SD semi-dry transfer cell; Bio-Rad, Hercules, CA, USA). The PVDF membrane was blocked with proteins and incubated overnight with primary antibodies. Nrf2 and NQO1 primary antibodies were purchased from ABclonal Technology (Woburn, MA, USA), UGT1A primary antibody was from GeneTex (Irvine, CA, USA) and HO-1 and GAPDH primary antibodies were obtained from Proteintech (Rosemont, IL, USA). The PVDF membrane was then incubated with goat anti-mouse or rabbit IgG-HRP secondary antibodies (Croyez Bioscience, Taipei, Taiwan) and the protein bands were visualised using a chemiluminescent substrate in an optical system (Fusion Solo 6S; Vilber, Collégien, France). Images were analysed using ImageJ software (*25*) and GAPDH was used as a standard control for normalisation and quantification.

### Statistical analysis

The experimental data were statistically analysed using SAS computer software (*26*). One-way analysis of variance (ANOVA) was performed, followed by Duncan's new multiple range test, to determine significant differences among the data.

## RESULTS AND DISCUSSION

### Thermal ageing leads to changes in the composition of black garlic in an electric cooker

**Fig. 1:**
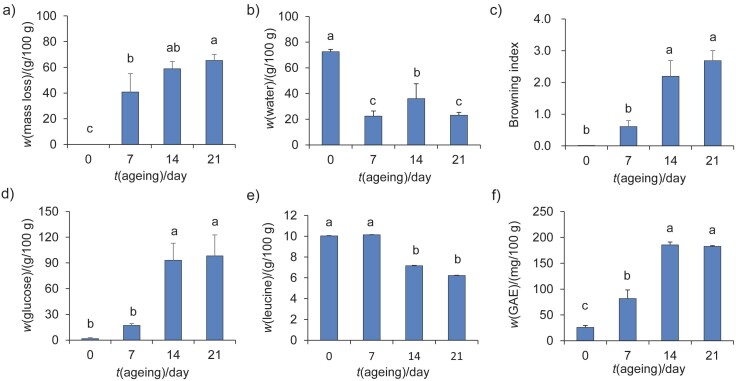
Effects of thermal ageing on: a) mass loss, b) water content, c) browning index, d) reducing sugar (sexpressed as glucose), e) free amino acids (expressed as leucine) and f) total phenols (expressed as gallic acid equivalents) content of garlic cloves after thermal ageing on days 7, 14 and 21. Different letters indicate significant differences (p<0.05) between the groups. Data are expressed as mean value±S.D., *N*=3

Black garlic has become increasingly popular on the market, which has led to an increased interest in preparing it at home. However, due to the lack of large-scale and professionally controlled ovens necessary for the traditional production method, households face limitations. Therefore, the aim of this study is to investigate the changes in the chemical composition that occur during the preparation of homemade black garlic using an electric cooker with a constant temperature of approx. 60 °C (Fig. S1 and Fig. 1). Our results indicate a significant mass loss of black garlic during the ageing process. In particular, a significant mass loss was observed after 7 days of ageing, reaching a maximum of approx. 60% after 14 and 21 days (p<0.05) (Fig. 1a). In addition, the water mass fraction in fresh garlic decreased significantly from 72.5 to about 22.5–36.1 g/100 g after 7, 14 and 21 days of ageing (p<0.05) (Fig. 1b). These results suggest that the observed mass loss in black garlic is primarily due to water evaporation. However, it is important to note that thermal ageing may also contribute to changes in the composition and subsequent mass changes of black garlic through the involvement of heat-sensitive volatile compounds and chemical reactions. According to previous research (*13*), fresh garlic is exposed to high temperatures (60–90 °C) and high relative humidity (80–90%) for a certain period of time during the production of black garlic. It has been found that temperatures above 90 °C can lead to bitterness, while temperatures below 60 °C lead to incomplete fermentation, resulting in a taste and colour intermediate between raw garlic and black garlic (*13*). The optimal water mass fraction for black garlic, which is reached after the ageing process is complete, is between 40 and 50 g/100 g and provides the desired texture and elasticity for consumption (*15*). A water mass fraction below 35 g/100 g can lead to dryness and poorer palatability (*15*). In this study, black garlic aged for 14 days in an electric cooker had a water mass fraction of approx. 36.1 g/100 g (Fig. 1b). The water content of the homemade black garlic is close to the recommended values, suggesting that the ageing process in the electric cooker can help achieve the desired water content for optimal taste and texture. The results suggest that homemade black garlic can have the same water content as commercially produced black garlic.

The Maillard reaction is a nonenzymatic browning process that occurs when sugars and amino acids react at high temperatures to produce unique flavours, colours and Maillard reaction products. In this reaction, the Amadori rearrangement leads to the condensation of sugars and amino acids, followed by the dehydration of sugars and degradation of amino acids (*27*). Subsequent condensation reactions can produce nitrogen-containing heterocyclic compounds that can affect the colour and aroma of the final product (*28*). The antioxidant properties of Maillard reaction products are also known (*27*). The characteristic black-brown colour and unique aroma of black garlic are caused by the Maillard reaction during the ageing process (*29*). In this study, we found a significant increase in the browning index during the thermal ageing of black garlic for a longer time (p<0.05), indicating the occurrence of Maillard reaction (Fig. 1c). The Maillard reaction could be responsible for the development of the distinctive colour and flavour of black garlic during ageing.

The Maillard reaction in garlic is triggered by high temperatures and high humidity, leading to an increase in intermediates and flavonoids, which may be responsible for the enhanced antioxidant properties of black garlic (*29*). We investigated the changes in reducing sugar content in this study and observed a consistent increase with longer ageing time (Fig. 1d). The reducing sugar content reached glucose mass fractions of approx. 93.2 and 98.3 mg/100 g after 14 and 21 days of ageing (p<0.05), respectively. The free amino acid content, expressed as leucine, on the other hand, showed a decreasing trend during the time and reached about 7.2 and 6.2 g/100 g after 14 and 21 days of ageing, respectively (Fig. 1e). According to these results, the use of an electric cooker for ageing black garlic leads to an increase in reducing sugars and a decrease in free amino acids. We also analysed the total phenolic content in black garlic at different ageing times (Fig. 1f). The total phenolic content, expressed as GAE, increased significantly after 7 days of ageing (p<0.05) and reached a value of approx. 81.8 mg/100 g. Subsequently, the total phenolic content continued to increase significantly after 14 and 21 days of ageing, reaching approx. 185.5 and 181.7 mg/100 g, respectively (p<0.05). These results indicate that the ageing of black garlic in an electric cooker leads to the accumulation of phenolic compounds.

### Thermal ageing of black garlic increases the DPPH radical scavenging ability and cellular antioxidant activity

**Fig. 2:**
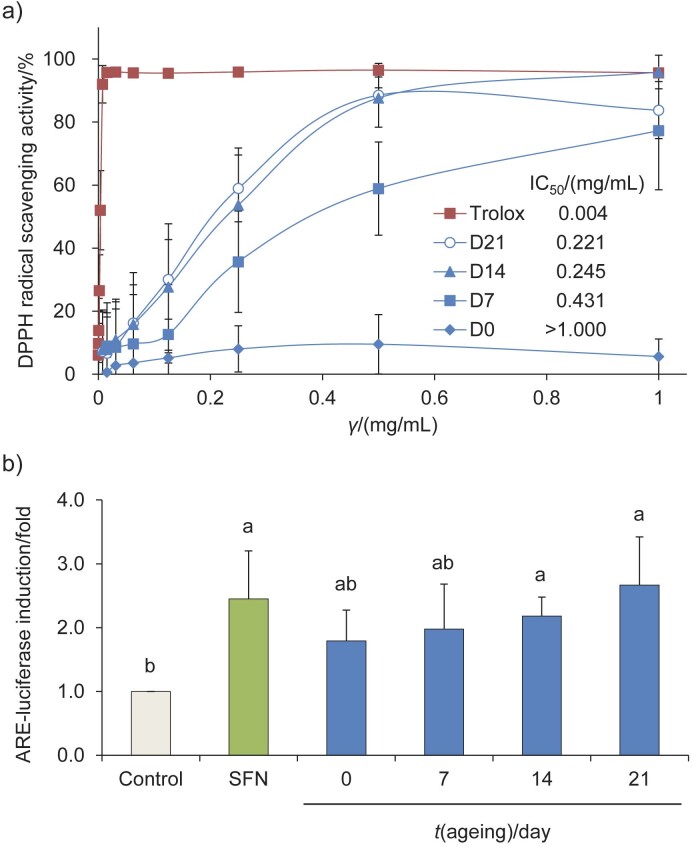
Effect of thermal ageing on DPPH radical scavenging and ARE-luciferase activities of black garlic extracts: a) DPPH radical scavenging activity and b) ARE-luciferase activity (expressed as protein equivalents). HepG2-C8 cells (10^5^ cell/mL) were seeded in a 12-well plate in DMEM medium for 24 h, followed by incubation in a new medium containing water extracts of black garlic after different thermal ageing (D7, D14 and D21) for an additional 24 h. Significant differences (p<0.05) between the groups are indicated by different letters based on the data (mean±S.D., *N*=3). SFN=sulforaphane

Black garlic is known for its diverse physiological activities and antioxidant potential (*13*). Processing methods may affect the antioxidant activity of garlic by converting alliin, a stable compound, to *S*-allylcysteine during ageing (*30*). Moreover, oral administration of 100 mg/kg black garlic in male Sprague-Dawley rats with ethanol-induced oxidative liver damage reduced the activities of AST, ALT, ALP and LDH in blood, while the expression of CYP2E1 in the liver was increased, indicating hepatoprotective effects (*31*). In this study, our aim was to investigate the enhanced biological activity of homemade black garlic with antioxidant potential. Our results suggest that the content of bioactive compounds, especially polyphenols, changes during ageing of homemade black garlic in an electric cooker. These changes may contribute to its physiological activities, including antioxidant capacity (Fig. 2). The results showed that black garlic aged for 14 and 21 days may have a higher total phenolic content and better DPPH radical scavenging ability (Fig. 2a). The concentrations (IC_50_) required for black garlic aged for 7, 14 and 21 days to scavenge 50% of DPPH radicals were 0.43, 0.24 and 0.22 mg/mL, respectively.

To evaluate the activation of cellular Nrf2 antioxidant/anti-inflammatory mechanisms, we examined the ARE-luciferase activity in HepG2-C8 cells treated with water extracts of black garlic (Fig. 2b). HepG2-C8 cells, which express the ARE-luciferase sequence plasmid, are commonly used for the evaluation of Nrf2 pathway induction (*23*). Sulforaphane (SFN), a known inducer of Nrf2 pathway, served as a positive control (*32*), and our study showed similar results. Water extracts (100 μg/mL without affecting cell growth) of black and fresh garlic resulted in a significant increase in ARE-luciferase activity compared to the control group (p<0.05). Although specific phenolic compounds were not analysed in this study, the increased content of phenolic compounds in black garlic likely contributes significantly to its antioxidant capacity. Future research should focus on analysing the specific phenolic compounds to gain a more comprehensive understanding of their role in the physiological activities of black garlic.

### Black garlic aged for 14 days received the highest overall preference

In the sensory evaluation, samples of black garlic aged for different times were assessed based on colour, aroma, texture and overall liking. Analysis of the statistical results (Table 1) showed that although no significant differences in preference for colour, aroma or texture were found, participants tended to prefer black garlic aged for 14 days in terms of overall preference (score of 5.88). This preference was also reflected in the ranking, with the black garlic aged for 14 days coming out on top. The 14-day-aged black garlic was praised by the participants for its soft texture, sweet and sour flavour and unique taste. Some participants did not like the aroma at first, but after giving it a second chance, they found it more acceptable. Regarding the sensory comments on the black garlic samples aged for the other two durations of time, fresh garlic was described as very spicy and pungent, while the black garlic aged of 7 days received comments such as a soy sauce-like taste and a less pronounced sweetness. The black garlic aged for 21 days was described as sour, soft, sweet and reminiscent of dried fruits. Some participants mentioned that they preferred the aroma after the first taste.

**Table 1:** Sensory analysis of black garlic

Sample random number	*t*(ageing)/day	Sensory evaluation^a^				Ranking^b^
		Colour	Aroma	Taste	Overall	
182	0	6.2±1.5	5.9±2.0	5.6±2.2	5.4±2.3	2.6±1.3
410	7	5.4±1.5	5.8±1.5	5.2±1.9	5.4±1.7	2.3±0.9
696	14	5.5±1.7	6.0±1.7	5.6±2.2	5.9±1.9	2.7±1.1
092	21	5.1±1.8	5.8±2.0	5.0±2.2	5.2±2.1	2.2±1.0

^a^Sensory evaluation of samples was carried out on a 9-point scale, with higher scores indicating a higher preference. In each analysis, there were no significant differences between the individual samples (p>0.05).

^b^After evaluating the samples, the impression scores were categorised on a scale from 1 to 4, with a higher score indicating a greater preference

Considering the total phenolic content, the DPPH radical scavenging ability and the activation of the Nrf2 antioxidant pathway in this study, black garlic aged for 14 and 21 days showed the potential to increase intracellular antioxidant capacity. However, sensory evaluation results revealed that the black garlic aged for 14 days was preferred. Further research should focus on investigating the potential protective mechanisms and effects of the water extract of black garlic aged for 14 days on liver cells against oxidative stress-induced damage.

### Black garlic water extract shows protective effects against H_2_O_2_-induced damage in HepG2 cells by activating the Nrf2 pathway

**Fig. 3:**
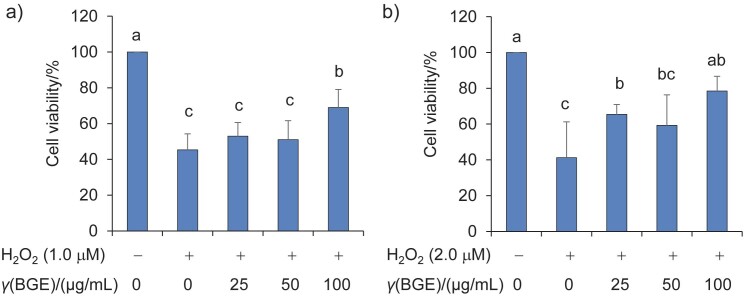
Effect of black garlic extract (BGE) on the growth of HepG2 cells against oxidative stress caused by H_2_O_2_. HepG2 cells (2·10^4^ cell/100 μL) were seeded in a 96-well plate with DMEM and incubated for 24 h. The cells were then treated with different concentrations of black garlic extract (thermal ageing on day 14) in a new DMEM for an additional 48 h. The medium with black garlic extract was then replaced with fresh DMEM containing: a) *c*=1.0 μM and b) 2.0 μM of H_2_O_2_, and treated for 8 h. Cell viability was determined using the MTS assay. Significantly different results (p<0.05) are indicated by different letters among the groups based on the data (mean±S.D., *N*=4)

The hepatoprotective potential of natural compounds is often evaluated using an H_2_O_2_-induced liver cell damage model (*33*). Furthermore, a selenium-enriched black garlic extract has been shown to protect against LPS/D-GalN-induced acute liver failure in rats by restoring metabolic balance, regulating intestinal flora and exerting antioxidant effects (*34*). In this study, we investigated the protective effect of the water extract of black garlic aged for 14 days against H_2_O_2_-induced HepG2 cell damage (Fig. 3). Fig. 3a and Fig. 3b show that H_2_O_2_ at concentrations of 1.0 and 2.0 μM significantly reduced cell viability to approx. 49 and 35%, respectively, compared to the control group (p<0.05). However, pretreatment of HepG2 cells with 100 mg/mL of black garlic extract for 48 h, followed by exposure to H_2_O_2_ for 8 h, significantly improved cell viability and protected them against cell damage caused by 1.0 or 2.0 μM H_2_O_2_ (p<0.05). These results suggest that black garlic extract can protect against H_2_O_2_-induced oxidative stress and cell damage in HepG2 cells.

**Fig. 4:**
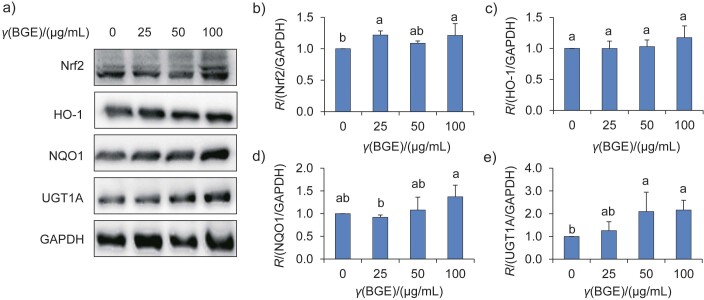
Effect of black garlic extract (BGE) on the protein expression of Nrf2-associated antioxidant enzymes in HepG2 cells. HepG2 cells (2·10^6^ cell/10 mL) were seeded in a 10-cm dish with DMEM and incubated for 24 h. The cells were then treated with different concentrations of black garlic extract (thermal ageing on day 14) in DMEM for an additional 48 h: a) Western blot analysis of Nrf2 and its downstream antioxidant enzymes, including HO-1, NQO1 and UGT1A. β-Actin was used as a loading control, b) Nrf2, c) HO-1, d) NQO1, and e) UGT1A protein expression levels were quantified by densitometry, with levels normalized to β-actin. Significant differences (p<0.05) among the groups are indicated by different letters based on the data (mean±S.D., *N*=3). *R*=number ratio

Numerous studies have shown that natural compounds have hepatoprotective effects, possibly through the activation of the Nrf2 pathway, including the upregulation of downstream antioxidant enzymes NQO1, HO-1 and UGT1A (*24*). In addition, research on industrial garlic peel waste has demonstrated its sustainability potential, with an increase in antioxidant and anti-inflammatory activities and activation of the Nrf2/HO-1/NQO1 pathway (*35*). In this study, black garlic water extract was initially found to be effective in inducing Nrf2-ARE-luciferase activity in HepG2-C8 cells (Fig. 2b). Therefore, further studies were conducted to test whether the water extract of black garlic aged for 14 days could increase the protein expression of Nrf2 and its downstream antioxidant enzymes (Fig. 4a). Compared to the control group (without treatment with black garlic extract), 100 μg/mL of black garlic extract was found to significantly increase the protein expression of Nrf2 (p<0.05) (Fig. 4b). Although the expressions of NQO1 and HO-1 were slightly increased by black garlic extracts, the differences were not significant (Fig. 4c and Fig. 4d). Regarding UGT1A, both 50 and 100 μg/mL of black garlic extract significantly increased the protein expression of UGT1A compared to the control group (p<0.05) (Fig. 4e). These results suggest that cells *in vitro* require longer treatment duration, such as 48 h, to activate antioxidant responses through mechanisms such as epigenetic modifications, leading to increased expression of Nrf2 target genes (*24*). Overall, these results suggest that black garlic water extract can activate the Nrf2 pathway and upregulate the expression of downstream antioxidant enzymes. This is the first study to establish that black garlic may have hepatoprotective potential *via* the activation of Nrf2-mediated antioxdiant mechanisms.

However, the significant results of the study are accompanied by limitations. The bioavailability and stability of the active garlic compounds during thermal ageing were not measured and normal liver cells were not used for comparison. In the future, it is important to explore these aspects and identify the specific phenolic compounds responsible for the observed antioxidant and hepatoprotective effects.

## CONCLUSIONS

The results of this study show that the duration of thermal ageing significantly affects the composition of black garlic, resulting from the Maillard reaction in fresh garlic. After 14 and 21 days of thermal ageing, black garlic showed a significant increase in total phenolic compounds and free radical scavenging capacity. The sensory evaluation revealed that the black garlic aged for 14 days was the most preferred by the participants. Further investigation revealed that the 14-day-aged garlic water extract upregulated the expression of Nrf2 and antioxidant-related enzymes, including NQO1 and UGT1A. This activation of the Nrf2 pathway may increase intracellular antioxidant capacity, leading to a reduction in H_2_O_2_-induced oxidative stress, liver cell damage and cell death. These results emphasise the potential of homemade black garlic prepared in an electric cooker as a functional food with enhanced antioxidant properties and hepatoprotective potential.
